# Simple and robust aqueous sheet jets in vacuum

**DOI:** 10.1107/S2052252526007013

**Published:** 2026-09-01

**Authors:** Patrick E. Konold, Adil Ansari, Daniel Westphal, Patrick Adams, Romain Letrun, Joana Valerio, Johan Bielecki, Roberto C. Alvarez, Szabolcs Bódizs, Katerina Dörner, Gabriel Ducrocq, Lukas Grunewald, Konstantin Kharitonov, Chan Kim, Marco Kloos, Anna Králová, Taru Larkiala, Michael Maihöfer, Dimitra Manatou, Robert Mehlig, Diogo V. M. Melo, Petra Meszaros, Leonardo Monrroy, Tokushi Sato, Robin Schubert, Peter Smyth, Egor Sobolev, Fabian Trost, Raphael de Wijn, Lena Worbs, Tej Varma Yenupuri, Tong You, Joachim Schulz, Richard Bean, Kartik Ayyer, María Agustina Domínguez Martin, Henning Kirst, Helmut Grubmüller, Sebastian Westenhoff, Richard A. Kirian, Filipe R. N. C. Maia

**Affiliations:** a Laboratory of Molecular Biophysics, Institute for Cell and Molecular Biology Uppsala University Box 596 75124Uppsala Sweden; b Department of Physics Arizona State University 550 E. Tyler Dr. Tempe AZ85287 USA; c European XFEL Holzkoppel 4 22869Schenefeld Germany; d Science for Life Laboratory, Center for Chemical Mechanisms of Life, Department of Chemistry – BMC, Biochemistry Uppsala University Uppsala Sweden; e Theoretical and Computational Biophysics Department Max Planck Institute for Multidisciplinary Sciences Göttingen Germany; f Max Planck Institute for the Structure and Dynamics of Matter 22761Hamburg Germany; g Departamento de Bioquímica y Biología Molecular, Campus de Excelencia Internacional Agroalimentario ceiA3 Universidad de Córdoba 14001Córdoba Spain; h Departamento de Genética, Campus de Excelencia Internacional Agroalimentario ceiA3 Universidad de Córdoba 14071Córdoba Spain; i Instituto Maimónides de Investigación Biomédica de Córdoba (IMIBIC), 14004Córdoba, Spain; Brookhaven National Laboratory, USA

**Keywords:** liquid sheet jets, aqueous thin films, in vacuum, XFELs

## Abstract

Free-standing thin liquid films offer a convenient way to probe matter in solution without a substrate, where a small sample thickness is required. The production of thin films in a vacuum environment is required for probes that cannot penetrate through a surrounding gas or in cases where the gas produces problematic background noise. Nozzles that produce continuously flowing liquid sheet jets have been employed recently for scattering and spectroscopy measurements at X-ray free-electron lasers (XFELs). However, their performance in vacuum with aqueous samples is complicated by high freezing susceptibility due to a strong evaporative cooling effect. Here, we introduce a vacuum-compatible gas-impinging liquid sheet jet nozzle capable of generating sub-100 nm aqueous films. The introduction of a second axisymmetric helium gas sheath surrounding the liquid orifice enables seamless startup and shutdown within a vacuum environment. We investigated the sheet geometry dependence under varying flow conditions and liquid viscosity and demonstrated its viability for X-ray scattering measurements at a high-repetition-rate XFEL source. Finally, we present potential strategies for reducing sample consumption. Our findings confirm that this nozzle exhibits simplified vacuum operation and may help promote the broader utilization of liquid sheet jets for several experimental applications.

## Abstract 2

A novel vacuum-compatible liquid sheet jet nozzle, utilizing a second gas sheath, successfully generates stable aqueous films under 100 nm in thickness, overcoming previous freezing limitations in vacuum environments. This development simplifies operations and is demonstrated to be viable for high-repetition-rate X-ray scattering measurements at X-ray free-electron laser sources, broadening the applications for studying matter in solution.

## 1. Introduction

High-speed liquid jets are a preferred means of sample delivery for several solution-phase experimental applications, particularly those at X-ray free-electron lasers (XFELs). These exist in several forms with varying sizes and offer rapid sample replenishment that is compatible with modern high-repetition-rate probe sources (Wiedorn *et al.*, 2018 ▸; Nazari *et al.*, 2020 ▸). The liquid sheet jet is an emerging variant that provides thin films reaching sub-100 nm thickness, a much larger target area, and flows that are more stable than cylindrical jets of comparable size (Koralek *et al.*, 2018 ▸; Konold *et al.*, 2023 ▸).

The sheet jet geometry has notable advantages for many experimental applications, in particular those with probes with short attenuation length in water, like electrons, mid-infrared or vacuum ultraviolet. For example, conventional cylindrical liquid jets with approximately micrometre thickness are unsuitable for some X-ray absorption spectroscopies because they exceed the attenuation length of soft X-rays in water and other liquids. Several groups have demonstrated the advantages of liquid sheet jets for X-ray absorption spectroscopy applications (Galinis *et al.*, 2017 ▸; Ekimova *et al.*, 2015 ▸; Barnard *et al.*, 2022 ▸; Gallo *et al.*, 2024 ▸). Ultrafast electron diffraction studies in solution also require thin films in vacuum because electrons, like soft X-rays, have a very shallow penetration depth in water and air. Water diffraction has been demonstrated at 0.6 Å resolution using ultrafast electron diffraction with liquid sheet jets (Nunes *et al.*, 2020 ▸; Crissman *et al.*, 2022 ▸). Liquid sheet jets may also be useful for mid-infrared vibrational spectroscopy, where attenuation lengths in water can be as low as 830 nm (Downing & Williams, 1975 ▸). Finally, novel model-free solution-phase imaging methods such as fluctuation X-ray scattering (Kurta *et al.*, 2017 ▸; Kam, 1977 ▸) and single-particle imaging (Seibert *et al.*, 2011 ▸) may be possible in solution since thin path lengths dramatically reduce solvent background and enable precise photon counting.

The above examples underscore the broad versatility offered by liquid sheet jets compared with cylindrical liquid jets. However, some issues need to be addressed in order to achieve their wider adoption: existing nozzle designs can be difficult to operate in vacuum and require high liquid flow rates, which make it practically impossible to study samples that are expensive or cannot be recirculated. Hence, there is a clear motivation for the development of robust liquid sheet jet sample-delivery platforms that offer extended data collection and seamless operation in beamline environments.

One approach for creating free-flowing liquid sheets involves colliding two Rayleigh jets (Taylor, 1959 ▸). This approach yields a sheet up to several millimetres long and micrometre thickness with an inverse relationship between thickness and the distance from the point of collision (Taylor, 1959 ▸; Hasson & Peck, 1964 ▸; Choo & Kang, 2002 ▸). A smaller and thinner sheet was demonstrated by Koralek *et al.* (2018 ▸) using an impinging gas to compress a Rayleigh jet into a sheet, resulting in film thicknesses down to 20 nm at a liquid flow rate of 150 µL min^−1^. We will subsequently refer to this type as a ‘gas-driven sheet jet’ (GDSJ) to more easily distinguish it from colliding-liquid variants. GDSJs were first shown to be stable while being probed with XFEL pulses at 120 Hz (Hoffman *et al.*, 2022 ▸; Koralek *et al.*, 2018 ▸) and later even at hundreds of kilohertz (Konold *et al.*, 2023 ▸).

Establishing aqueous sheet jets in vacuum is challenging due to the strong evaporative cooling effect, as any stray liquid that contacts the nozzle surface in vacuum can lead to catastrophic freezing events that require a vent cycle of the chamber. This effect was exacerbated in early designs where the meniscus lies on the nozzle exterior and is directly exposed to the low-pressure environment. Such delays are very costly in terms of both time and money, especially within beamline settings. An additional complication, given their intrinsically high liquid flow rates, is the growth of stalagmites comprising ice or non-volatiles that can disrupt jetting and damage the nozzle. Therefore, complex sample-catching strategies are generally used to capture the liquid emitted from the nozzle (Barnard *et al.*, 2022 ▸; Fujiwara & Midorikawa, 2025 ▸).

In this paper, we introduce a novel 3D-printed GDSJ nozzle design that eliminates catastrophic icing events, allowing for reliable in-vacuum startup and shutdown with liquid samples that are prone to freezing. We characterize the resulting sheet jets according to liquid and gas flow rates, nozzle geometry, and liquid viscosity. We also evaluate nozzle performance within a beamline setting by conducting hard X-ray scattering measurements using a high-repetition-rate XFEL source [SPB/SFX (Single Particles, Clusters and Biomolecules, and Serial Femtosecond Crystallography) instrument at the European XFEL (EuXFEL)]. Finally, we discuss potential strategies to reduce sample consumption.

## 2. Methods

### 2.1. Nozzle design

**Figure 1:**
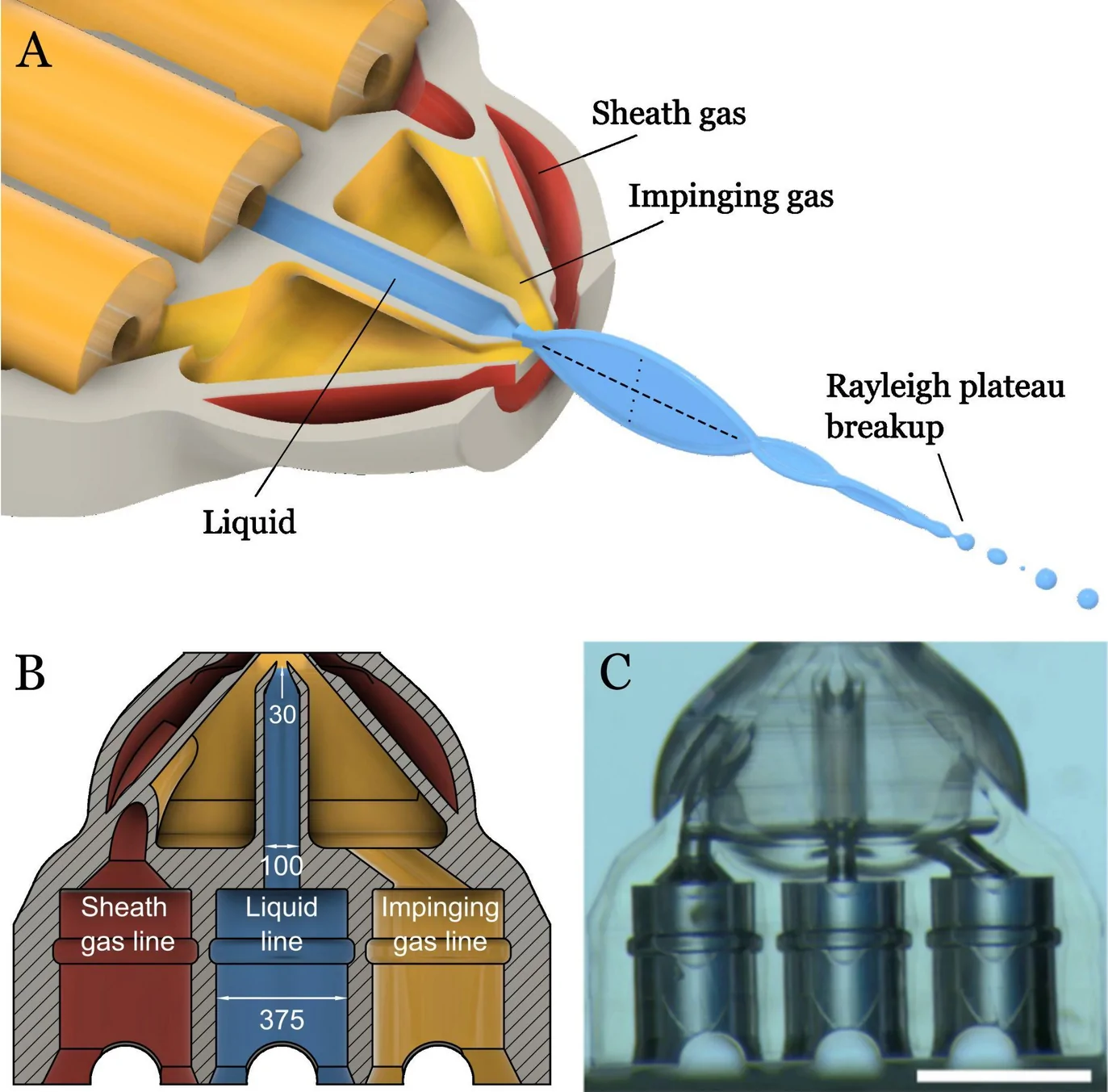
(*A*) Schematic drawing of our vacuum compatible GDSJ nozzle with color-coded labeling used to highlight internal gas and liquid channels. The length (- - -) and width (···) of the primary liquid sheet are defined. The jet speed can also be inferred by using droplets formed from Rayleigh-plateau instability. (*B*) CAD model of the GDSJ nozzle developed by the ASU group with channel dimensions in micrometres. The central inlet delivers the liquid sample, while the impinging gas line forms the sheet, and the sheath gas ensures stable operation in vacuum conditions. (*C*) Microscope image of the 3D-printed nozzle (white scale bar represents 0.5 mm). The Uppsala nozzle design is provided in Supporting Fig. 1. Both designs are publicly available online.

Our nozzle design follows from earlier GDSJ variants (Konold *et al.*, 2023 ▸; Koralek *et al.*, 2018 ▸). In this configuration, a Rayleigh jet is separated by colliding gas streams (80° impingement angle), resulting in pinching of the jet into two sheet arms that are connected by a thin film [Fig. 1 ▸(*A*)]. The largest modification compared with the earlier versions is the addition of a second gas channel that surrounds the nozzle outlet. This additional sheath gas surrounds the impinging gas and creates a local region of increased pressure to isolate the liquid emerging from the inner nozzle orifice from the surrounding vacuum environment. This enables fast and reliable jet startup and shutdown in vacuum since extraneous liquid harmlessly falls away as droplets rather than icing at the nozzle surface. This is akin to a gas dynamic virtual nozzle (GDVN) operation in vacuum, which is possible because the liquid meniscus is recessed in the nozzle where the gas pressure is high enough to prohibit ice formation (DePonte *et al.*, 2008 ▸).

Another important design modification is the reconfiguration of the inner channel geometries to further accommodate reliable in-vacuum startup and to help mitigate channel clogging. As illustrated in Fig. 1 ▸(*B*), flow resistance was significantly reduced by expanding channel dimensions within the body of the nozzle, which are then constricted just above the output orifice. In this way, overall line pressures are lower compared with previous designs that contained long and narrow inner channels (Konold *et al.*, 2023 ▸). A convenient side effect is that occasional obstructions within the liquid channel may be removed by ramping the line pressure. This modification also improves the fabrication quality of 3D-printed nozzles by allowing more efficient solvent penetration during the development step.

### 2.2. Nozzle fabrication by 3D printing

Separate nozzle variants were developed in parallel by groups at Arizona State University (ASU) and Uppsala University using different two-photon polymerization 3D-printer systems. This allowed us to test if the printer setup (*e.g.* resin or printing protocol) dramatically influenced nozzle performance. The overall nozzle designs were similar with minor deviations in the bulk structure. To accelerate the printing process, the overall nozzle volume was optimized for efficient fabrication, resulting in a print time of ∼45 min and 4 h for the UpNano NanoOne (Uppsala) and Nanoscribe (ASU) systems, respectively. A more detailed protocol for each configuration is provided in the supporting information. A development time of more than 12 h was necessary to ensure the complete removal of unpolymerized resin. The nozzles were then washed with iso­propanol and stored until final assembly.

Prior to assembly, the nozzles were removed from iso­propanol and dried. Fused silica capillaries (outside diameter OD of 360 µm and inside diameter ID of 150 µm, Polymicro TSP100375) were connected to the nozzle inlets with 5 min ep­oxy glue and attached to a nozzle rod connector as demonstrated by Nazari *et al.* (2020 ▸). This system is compatible with a standard nozzle rod load-lock system for convenient vacuum operation (Weierstall *et al.*, 2012 ▸).

### 2.3. Nozzle operation in offline test chamber

The liquid flow rate was controlled by a high-performance liquid chromatography (HPLC) pump (Shimadzu LC-20 AD), and the impinging and sheath gas flow rates were controlled by electronic gas flow regulators (Bronkhorst EL Flow). A sufficiently large catcher well of at least 25 cm depth was used to mitigate problems of stalagmite build-up. During stable operation, water droplets resulting from the sheet jet can accumulate on surfaces below to form an ice stalagmite. These grow quickly (>1 cm s^−1^), can reach several centimetres in length, and will disturb jetting behavior without a proper mitigation strategy. An alternative solution is to use a tilted rotating plate or chopper installed at an intermediate distance (15–20 cm) below the nozzle.

Before inserting the nozzle into vacuum, the sheath gas flow was started (∼50 mg min^−1^) to avoid ice formation from residual water within the channels. The water was held back using a fast-switching valve (Rheodyne 7000L) while pressure was built up to 1 kpsi using an HPLC pump set to the desired liquid flow rate (∼150 µL min^−1^). Once the desired pressure was reached, the Rheodyne valve was switched to initiate liquid flow and create a Rayleigh jet. Upon establishing stable jetting, the impinging gas flow was slowly ramped to the desired level (∼15 mg min^−1^) to yield a sheet jet. In principle, the sheath gas may be turned off once stable jetting is achieved. However, this comes with the risk of icing due to unexpected perturbations in the liquid flow (*e.g.* partial clogging, *etc*).

To shut down the sheet jet in vacuum, the sheath gas flow was resumed if not already active. Next, the impinging gas was reduced to a minimal level to prevent liquid backflow and stray jetting. Finally, the liquid flow may be stopped. The sheath gas was kept running to prevent icing from residual liquid in the lines while removing the nozzle from the vacuum environment. If multiple samples need to be probed, a switching valve is used to swap between reservoirs. When making the switch, the impinging gas flow was shut off while ensuring the sheath gas was flowing during the crossover period.

### 2.4. Liquid sheet jet optical characterization

**Figure 2:**
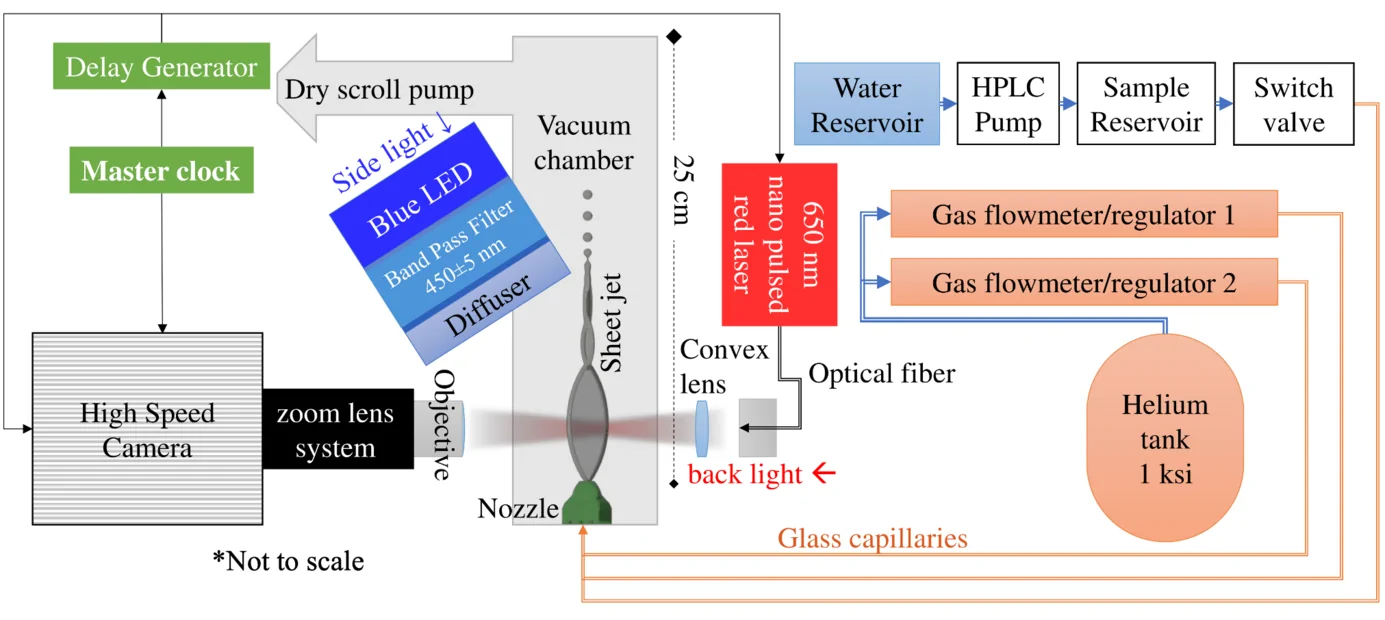
The optical imaging setup used to investigate liquid sheet jets in vacuum. Both continuous and pulsed illumination are available (diffused 450 nm LED or a 650 nm nanosecond dual pulsed laser). The side light was used for reflection interferometry, while the back light was used for general jet imaging.

An imaging setup equipped with a custom nozzle testing station (Nazari *et al.*, 2020 ▸), as shown in Fig. 2 ▸, was used to extract sheet jet geometric parameters. The jet was illuminated with a monochromatic 450 nm LED backlight (Thorlabs M455L3), narrowed with a band-pass filter (Thorlabs FBH450-10) and smoothed with a diffuser (Thorlabs DG 10 220 MD). Images were acquired using a high-speed camera (Photron SA5) with an optical microscope system containing a zoom lens (Navitar 12×) and a 10× long working distance objective. A separate fast illumination source containing dual nanosecond laser pulses (Thorlabs NPL52C, 550 ns delay, frame straddled) was used to compute the jet speed. The pixel separation using cross-correlation of the images post-Rayleigh-plateau breakup was used to obtain the jet speed. To measure sheet thickness, the near-monochromatic light source was positioned as a side light with the sheet jet angled 45° to reflect light towards the camera.

**Figure 3:**
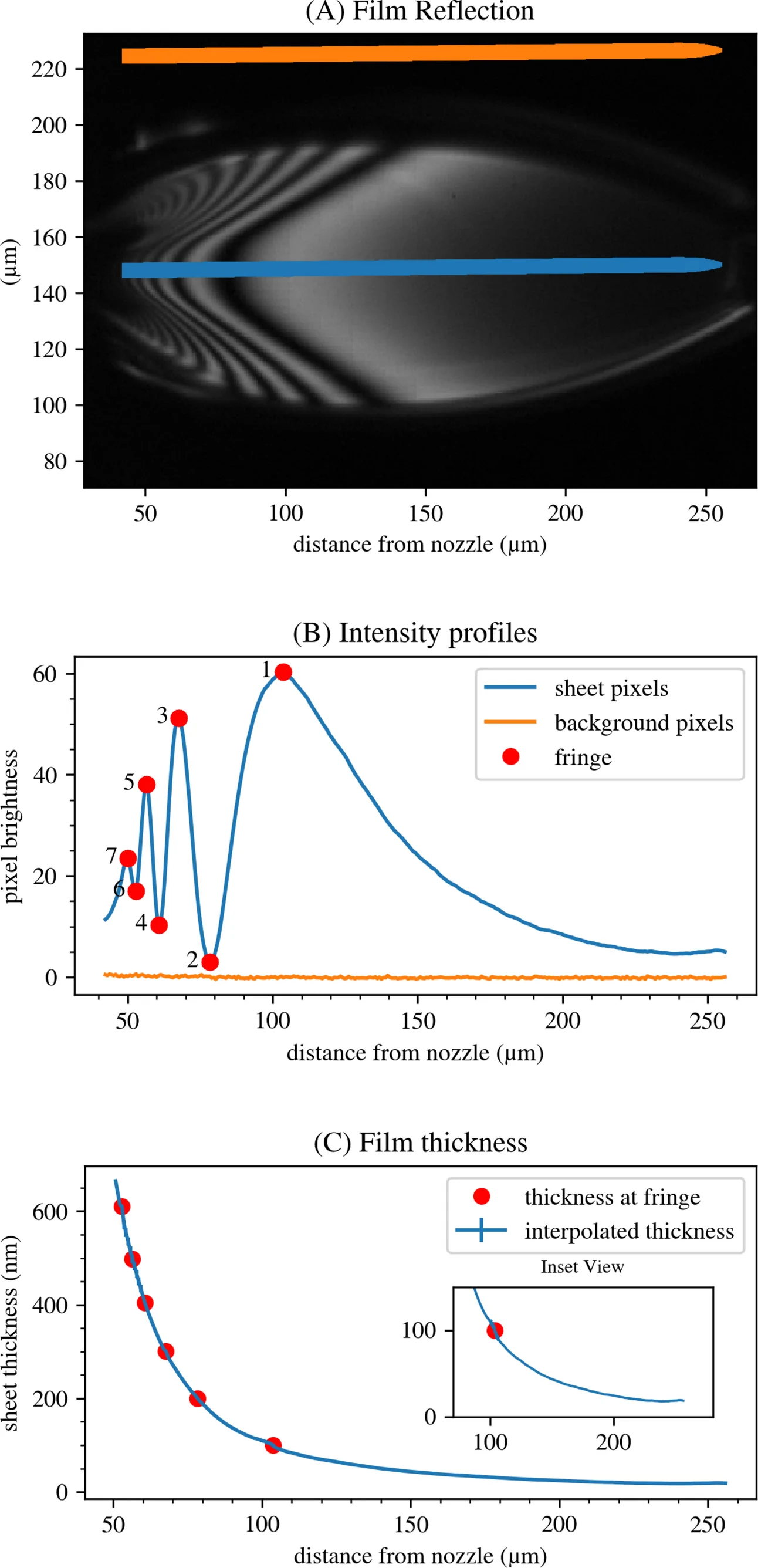
Measurement of the sheet thickness from the thin-film interference fringe pattern. (*A*) Image of a sheet reflecting diffused monochromatic light with the areas used to define the centerline and background marked in blue and orange, respectively. (*B*) Pixel brightness and background as a function of the distance to the nozzle. Local extrema representing light and dark fringes are marked in red. (*C*) Thickness derived from the brightness measurement.

The sheet thickness was derived from the fringe pattern within the reflected light using known relationships for thin-film interference. A full derivation is provided in the supporting information. Assuming near-monochromatic light of wavelength λ with collimated illumination of constant intensity 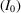, with uniform index of refraction (*n*) and negligible higher-order reflections, the normalized reflectance (*R*) can be expressed as a sinusoidal function of the thickness (*t*):
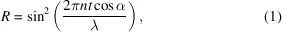
where α is the angle of refraction in a liquid sheet (see the supporting information for a more detailed derivation). *R* = 1 corresponds to maximum reflectance (light fringe) resulting from constructive interference, while *R* = 0 corresponds to minimum reflectance (dark fringe) due to destructive interference. The sheet thickness corresponding to the *m*th fringe is
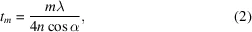
where even *m* values correspond to dark fringes and odd *m* values correspond to bright fringes. Given a pixel value *p_m_* at the *m*th fringe, one can interpolate the thickness as a function of the pixel brightness value *p* as
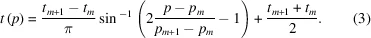
*P*_0_, which corresponded to thickness *t*_0_ = 0 nm, was assumed to be the average of background pixel brightness, as shown in Fig. 3 ▸(*B*). The position and pixel brightness of the fringes were determined using a local extrema-finder. The overall fitting results are described in Fig. 3 ▸. Laser speckle can complicate this phase unwrapping. While a spatially filtered laser beam could eliminate the speckle, a monochrome diffused light is preferred for the ease of sheet alignment and film-thickness extraction.

### 2.5. Liquid sheet jet sample injection at EuXFEL SPB/SFX

The printed sheet jet nozzles were mounted to the standard liquid injector rod provided by the EuXFEL Sample Environment group. The rod was assembled as follows. An 1/8 inch OD stainless steel tube was first glued to the capillaries ∼5–10 mm above the nozzle. This tube was fed through a 10–32 PEEK fitting (IDEX) that was fastened into a custom-built stainless steel nozzle adaptor. The individual capillaries (length ∼20 cm) were then connected to 500 µm ID PEEK lines that were fed through the entire length of the rod using IDEX sleeve fittings. By making these connections at the bottom of the rod, rather than at the top as is customarily done, one can avoid using long capillaries with smaller ID (∼150 µm) over this distance (>2 m), and hence the overall line pressure is significantly reduced. This configuration enables the use of high-precision flow regulators that are essential for maintaining sheet jet stability. Photographs of this assembly are provided in Supporting Fig. 2.

Liquid and gas were delivered to the nozzle as previously described (Vakili *et al.*, 2022 ▸). Briefly, liquid reservoirs were connected to the nozzle inlets via PEEK tubing (IDEX, 0.250 mm ID). Multiple sample reservoirs were connected in parallel to facilitate fast sample switching using a high-speed electronic valve (Rheodyne). Liquid flow was regulated using an HPLC pump (Shimadzu LC-20AD), while helium gas flow was regulated with an electronic pressure regulator (Proportion-Air QPV). Liquid and gas flow rates were monitored with in-line flow meters (Sensirion and Bronckhorst, respectively).

Alignment of the nozzle tip with respect to the interaction region was carried out by manipulating the position of the injector rod using motorized stages. This placement was aided by visualization with the side-view microscope camera illuminated with the EuXFEL femtosecond laser coupled into the sample chamber via a fiber bundle laser and synchronized with the X-ray pulse (Koliyadu *et al.*, 2022 ▸; Palmer *et al.*, 2019 ▸). Tuning to the X-ray focus was guided by maximizing the visible radiation-induced damage, together with monitoring the diffracted X-ray scattering intensity.

### 2.6. SPB/SFX beamline configuration

The data were collected at the SPB/SFX instrument of the EuXFEL in March 2024, under the proposal p5376 (Mancuso *et al.*, 2019 ▸). The XFEL produced bunched trains at 10 Hz with intratrain pulse repetition rates between 0.141 and 1.13 MHz. The photon energy was 6000 eV, ∼2.07 Å. From previous measurements, the focal spot was estimated at around 300 × 300 nm. The X-ray pulse energy was measured by a gas monitor detector upstream and was close to 2 mJ. With this beamline configuration and photon energy, the beamline transmission between the gas monitor detector and the interaction region is estimated to be 65%. The AGIPD 1M detector was 1 m downstream from the interaction region (Allahgholi *et al.*, 2019 ▸). The experiment was monitored online with *Hummingbird* (Daurer *et al.*, 2016 ▸).

## 3. Results

**Figure 4:**
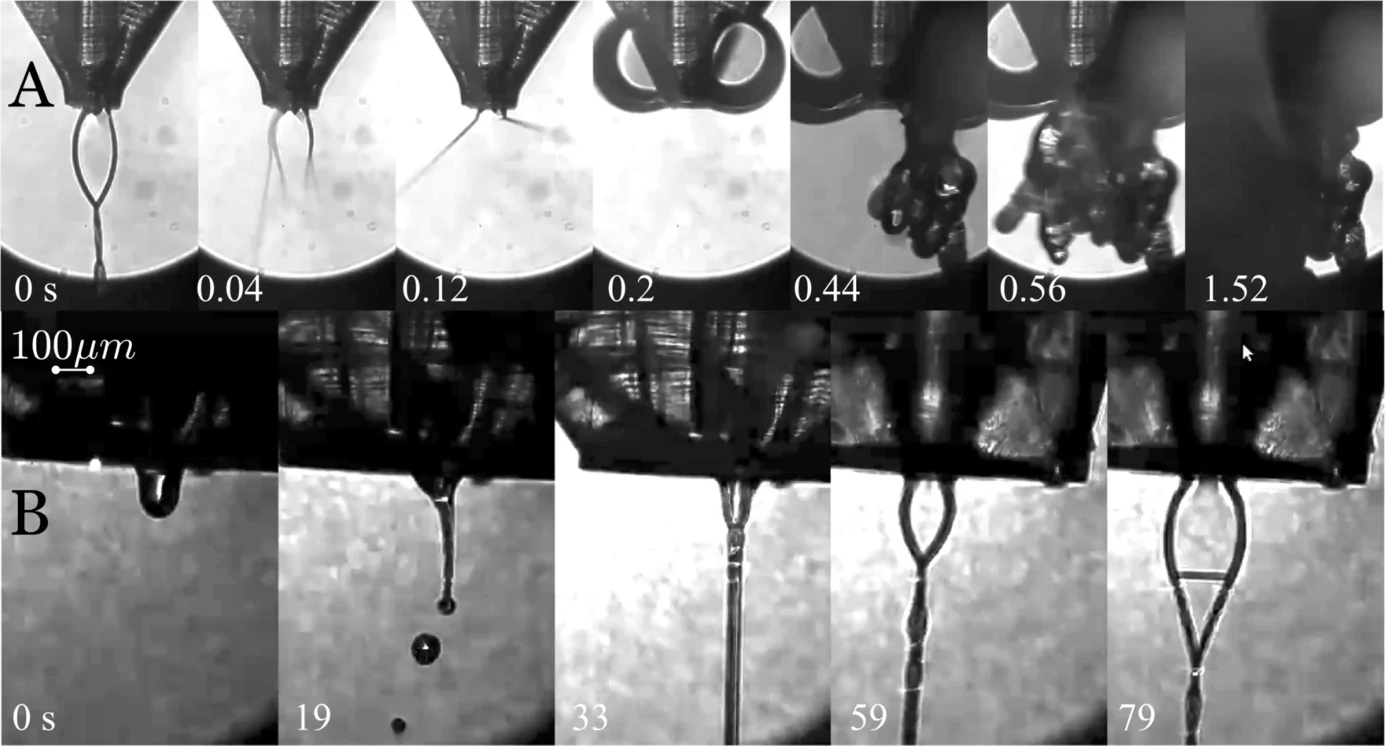
(*A*) Failed startup of a water sheet jet in vacuum using a conventional GDSJ nozzle. The icing process is typically irreversible and begins within one second of flow onset. (*B*) Successful startup of an aqueous sheet jet utilizing our modified nozzle employing a sheath gas.

The erratic vacuum startup behavior using a conventional sheet jet nozzle is shown in Fig. 4 ▸(*A*). Ice formation occurs rapidly (<1 s), caused by water supercooling and nucleation on the surface of the 3D-printed nozzle. This problem is eliminated by using an additional sheath gas channel that surrounds the liquid orifice. Fig. 4 ▸(*B*) illustrates the stages of successful jet formation: dripping, Rayleigh jetting, initial gas impingement and sheet formation. The timescales here are intentionally prolonged to illustrate the nozzle stability in vacuum and do not reflect the fastest possible startup time (<5 s in most cases).

**Figure 5:**
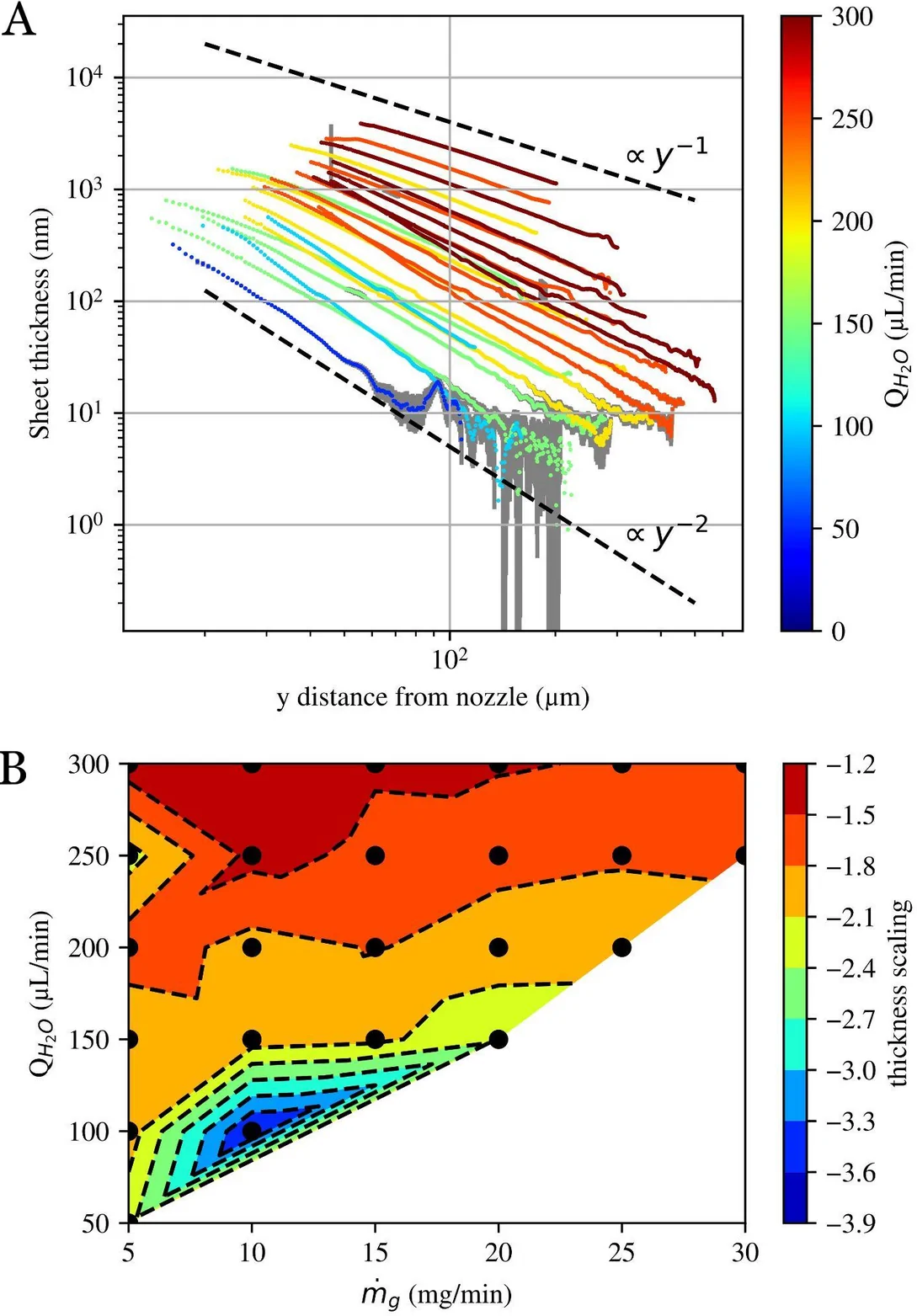
(*A*) Overlay plot of centerline sheet-thickness profiles for a range of liquid (water) flow rates. The gray shaded area corresponds to the standard deviation of the thickness. For each liquid flow rate, the colored plot sequences with the largest measured thicknesses correspond to gas flow rate of 5 mg min^−1^, whereas the plots with the smallest thicknesses correspond to 30 mg min^−1^ of gas flow. (*B*) Value of *c* derived from fitting equation (5) to thickness profiles varying as a function of liquid and gas flow rate.

The sheet jet geometry and velocity dependence were investigated. The water flow rate was varied from 50 to 300 µL min^−1^ in 50 µL min^−1^ increments. Similarly, the helium impinging gas flow rate varied from 5 to 30 mg min^−1^ with increments of 5 mg min^−1^. The sheath gas flow rate was held fixed at 50 mg min^−1^. All points with a flow ratio below 10 liquid:gas (µL mg^−1^) were not recorded because the sheet approaches the breakup threshold. The full results are provided in Supporting Fig. 7; however, the general trends are summarized in Table 1 ▸. We also calculated the Ohnesorge number (Oh) defined as a function of viscosity (μ), surface-tension coefficient (γ), liquid density (ρ) and thickness (*t*):
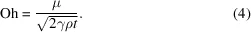
As expected, the sheet thicknesses along the centerline of the jet decreased as a function of distance from the nozzle, as shown in Fig. 5 ▸(*A*). The thickness data were fit to the following model:
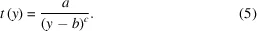
Here, *y* is the distance from the nozzle along the centerline of the sheet, while *a* and *b* are fitting parameters that represent amplitude scaling and an offset in the nozzle distance, respectively. The scaling law fell between *y*^−1^ and *y*^−2^ and, in general, this exponent (*c*) was observed to decrease with lower liquid flow rates and higher gas flow rates. Lastly, the long-term nozzle behavior in vacuum was evaluated over a 30 min period with liquid and impinging gas flow rates of 150 µL min^−1^ and 8 mg min^−1^, respectively. The reflectance profile did not significantly deviate over time (30 min), as shown in Supporting Fig. 4. The thickness was measured to be 55 ± 4 nm, including outlier time points.

### 3.1. Nozzle operation at EuXFEL SPB/SFX

Pilot beamline measurements were carried out at the EuXFEL SPB/SFX instrument. The explosion of the water sheet jet upon interaction with the ultrabright nanofocused XFEL pulses was recorded at high repetition rates. The behavior was similar to our previous report of iso­propanol sheet jets exposed to an XFEL beam (Konold *et al.*, 2023 ▸). The resulting puncture is caused by the intense localized heating and ionization effects from the X-ray absorption, leading to rapid vaporization or cavitation in the sheet. A region of plasma is visible at the interaction point. As observed with iso­propanol, the explosion did not impact the overall sheet geometry above the interaction region and only became catastrophic when the expansion front reached the lower sheet arms. Frame grabs and stroboscopic movies of the jet explosion captured with the side microscope camera are provided in Supporting Figs. 5 and 12. Moreover, the overall jetting behavior was unperturbed upon exposure to the ultra-bright XFEL pulse. Extended data-collection intervals for several hours were carried out with no clogging or freezing events for a range of samples, including pure water, protein complexes in pH buffer, and nanoparticle suspensions. This included several shutdown and startup sequences to allow for changing sample reservoirs.

The jet stability was assessed by normalizing the detected scattered X-ray intensity with the incoming X-ray gas monitor response. These data, together with our previous measurements (proposal p3046) involving GDVNs and iso­propanol GDSJs, are given in Supporting Fig. 11. We found that the vacuum-compatible nozzle exhibited twofold higher stability compared with our previous GDSJ nozzle variant.

### 3.2. Sheet jet viscosity dependence

One potential avenue to reduce sample consumption is to lower the overall liquid flow rate. In practice, there is typically a minimum jet size that can be used for a given experiment, which puts a lower bound on the liquid flow rate required. For X-ray scattering measurements with GDSJ nozzles, the minimum acceptable sheet geometry corresponds to a liquid flow rate of roughly 150 µL min^−1^. However, it may be possible to exploit the effect of liquid viscosity, which is known to increase overall sheet size, to push this limit lower.

**Figure 6:**
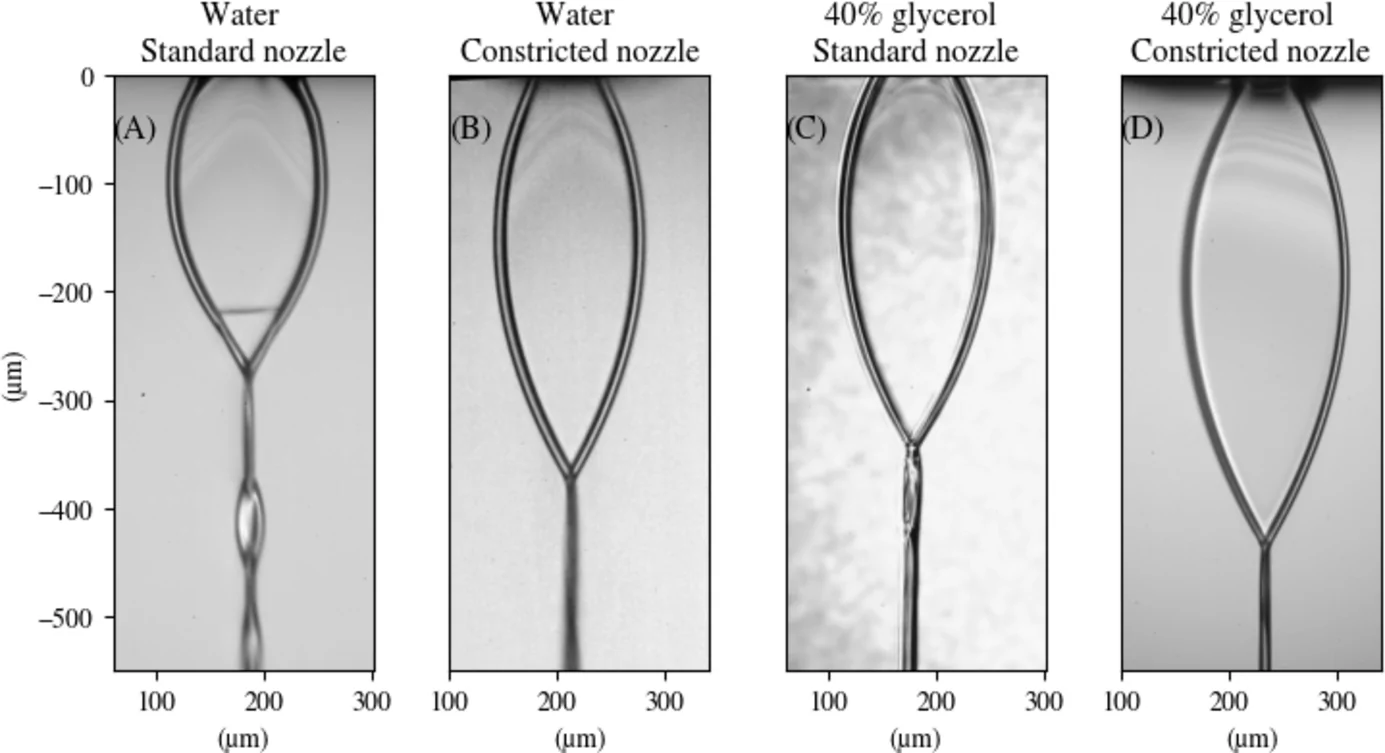
Sheet size dependence versus liquid viscosity. A constant liquid flow rate of 150 µL min^−1^ was used for all cases. (*A*) A standard nozzle with pure water yields a maximum length of 220 µm. (*B*) A constricted nozzle with orifice reduced from 30 × 30 µm (length × width) to 20 × 20 µm extends the length to 370 µm. (*C*) A standard nozzle with 40% glycerol, which raises the viscosity of the solution, increases the length to 350 µm. (*D*) The greatest size enhancement was achieved by combining 40% glycerol with orifice constriction, which yields a length of 450 µm.

We performed offline experiments to explore the effect of sample viscosity (Fig. 6 ▸). To begin, a sheet jet composed of pure water and generated with a standard nozzle was found to have a maximum length of 220 µm. By increasing the viscosity (40% glycerol), the sheet is lengthened to 350 µm [Fig. 6 ▸(*C*)]. Since liquids are incompressible, this effect may be even further extended by raising the jet velocity. This was achieved by using a modified nozzle with a constricted liquid orifice reduced from 30 × 30 to 20 × 20 µm (length × width) that yielded a sheet length of 450 µm. Overall, this represents a greater than twofold increase in length.

Given the dramatic increase in sheet length shown in Fig. 6 ▸(*C*), further investigation was necessary. The liquid flow rate of a 40% glycerol solution was increased while the gas flow rate was optimized to achieve maximum sheet length before the jet structure broke apart. As shown in Supporting Fig. 6, the sheet length extends from 150 to ∼500 µm when the flow rate is increased from 50 to 120 µL min^−1^. Similarly, the width increases from 80 to ∼200 µm, respectively. Hence, by employing a more viscous sample solution, in principle, one could operate at less than twofold lower liquid flow rate and achieve comparable sheet dimensions relative to neat water. This would correspond to ∼70 µL min^−1^ for typical operating conditions. While the overall liquid flow rate is reduced, more impinging gas is needed to drive a viscous sheet jet, up to 28 mg min^−1^.

## 4. Discussion

The above results demonstrate the robust performance of our novel sheet jet nozzle in a vacuum environment. Reliable operation in vacuum is not merely a convenient experimental benefit, but one that may lead to significant cost savings and potentially expand user access when deployed in competitive beamline settings. During our beamline investigation at EuXFEL SPB/SFX, the additional sheath gas load was found not to drastically compromise vacuum levels that settled in the 10^−5^ mbar range in the main chamber. This is not unreasonable considering the total gas load is similar to a GDVN (~60–70 mg min^−1^). However, we found that the catcher pressure intermittently reached borderline values when running at high liquid flow rate (up to 250 µL min^−1^). This may be overcome by employing relatively straightforward cryotrapping approaches. Depending on experimental requirements, heavier sheath gases may be employed (*e.g.* N_2_) that might alter the freezing threshold. Helium is preferable for X-ray scattering measurements since it offers the lowest background contribution.

In general, our offline investigations revealed flow-dependent sheet geometry consistent with previous reports of liquid-colliding jets. Increasing gas flows led to longer, wider, and also thinner, sheets, irrespective of absolute or size-relative position. The sheet width was found to be independent of the liquid flow rate, though it is unclear if this trend holds with different nozzle geometries. It was also observed that increased viscosity allows for increased physical dimensions, likely due to increased fluid stability. The required gas flow for creating a viscous sheet jet is considerably higher than for pure water. The influence of the additional sheath gas was also examined and found not to perturb the sheet structure under normal operating conditions. However, it may contribute to a slight impingement effect under high gas flows.

We found that optical thin-film interferometry is a convenient and cost-effective means of measuring sheet jet thickness down to tens of nanometres. In this way, accurate thickness profiles of the entire flat section may be extracted in a single-shot fashion with minimal hardware and computational effort. An important caveat with this method is that the zero-order fringe must be correctly assigned to retrieve accurate thickness values. We also acknowledge that other methods of thickness determination, such as optical or infrared absorption, fluorescence, or polarization techniques, are valid alternatives.

The sheet thickness along the centerline of the jet as a function of distance from the nozzle was found not to obey the traditional scaling law of *y*^−1^ under all flow conditions (Choo & Kang, 2001 ▸). With increased gas flow, it is observed that the scaling is closer to *y*^−2^. Since continuity must be conserved, this would imply either that the gas is continuing to impinge on the film well beyond the nozzle outlet or that the film is accelerating due to impinging gas shear or both despite rarefaction of helium. Typically, micro- and nano-jets expelled in vacuum by a GDVN tend to have no acceleration after a few tens of micrometres from the nozzle orifice in vacuum (Karpos *et al.*, 2024 ▸). The reason for film acceleration may be that for a sheet jet nozzle, the gas flow is more localized, unlike axisymmetric flow focusing, imparting more momentum on a liquid film that has a considerable surface area and is also significantly thinner. Another explanation for *y*^−2^ scaling might be evaporation from the thin film.

The sheet flatness was evaluated by comparing the thickness profiles of horizontal slices at varying distances from the nozzle orifice. Here, we find a stronger curvature compared with earlier nozzle variants (Konold *et al.*, 2023 ▸). Since the channel dimensions near the outlet orifice are similar in both designs, it is somewhat surprising to see such a large variation. This underscores the importance of applying computational approaches to better understand the mechanism of gas–liquid impingement. While numerical modeling of these systems, especially within a vacuum environment, is exceedingly difficult, such knowledge may be critical for the development of next-generation nozzles with expanded functionality.

Film thicknesses approaching 10 nm for varying flow conditions were achieved [Fig. 6 ▸(*A*)]. This minuscule thickness is smaller than previously reported for GDSJ nozzles and implies only ∼40 water molecules from face to face (Koralek *et al.*, 2018 ▸; Konold *et al.*, 2023 ▸). The sheet thickness was found to change slightly over time, but can be tracked, and the uncertainty is reasonably small.

Both the thickness stability and sheet geometry are particularly sensitive to the means of driving liquid and gas flow. For this reason, robust regulation of both liquid and gas flow rates is required in order to suppress undesirable fluctuations. In this study, this was achieved by using an HPLC pump and high-precision commercial gas flow controllers (Bronckhorst and Proportion-Air QPV). While the deviations observed here were small, worse performance might occur with poor instrument maintenance or improper solution handling (*e.g.* inadequate sample degassing). In particular, these fluctuations may become burdensome when probing the thinnest part of the jet, near the lower rim. Deviations in the jet position may lead to inadvertent exposure of the much thicker rim section, causing potentially harmful jet streaks on the detector. Accordingly, careful deployment of high-precision flow controllers and sturdy nozzle mounting are suggested when operating in such risky configurations.

### 4.1. Approaches to reduce sample consumption

Despite the dramatically improved vacuum performance demonstrated here, further challenges exist towards the optimization of liquid sheet jet sample delivery. A potential strategy of exploiting the effect of increased jet velocity combined with a higher sample viscosity was found to lower the overall effective liquid flow rate by more than twofold. While this reduction is meaningful, it comes with certain drawbacks. For example, glycerol is not compatible with all samples and higher carbon content within the solvent may decrease contrast and complicate measurements. Furthermore, constricting the nozzle orifice may increase the risk of clogging for certain samples. Thus, we find that this is an imperfect means to reduce overall sample consumption.

Future efforts will focus on the generation of sheet jets composed of two liquids, where the sample flows together with a second carrier liquid (*e.g.* aqueous buffer). Given that much of the liquid volume within the sheet jet passes through the interaction region uninterrogated, this fraction essentially amounts to wasted material without the presence of a robust recirculation system. Thus, the aim is to localize the sample fraction inside the central sheet region, while confining a sacrificial liquid within the sheet arms. This approach, when combined with segmented flow schemes, may significantly reduce sample quantities.

## 5. Conclusions

Liquid jets are indispensable tools for investigating molecular systems in the solution phase. Sheet jets have specific advantages given their flat geometry, large target area, and nanoscopic thickness compared with conventional cylindrical jets. In this study, we introduced a novel 3D-printed sheet jet nozzle design that overcomes previous challenges with vacuum operation and enables prolonged data-collection intervals and accumulation of large datasets. Our findings highlight the importance of further research into nozzle optimization, as well as the versatility offered by 3D printing to rapidly prototype new designs. These devices have the potential to outperform existing technologies and open new methods for enhanced solution-phase experimental investigations.

## Supplementary Material

Supporting information. DOI: 10.1107/S2052252526007013/if5007sup1.pdf

## Figures and Tables

**Table 1:** The effect of the gas and liquid flow rates on the liquid sheet jet physical dimensions and speed

Liquid sheet property	Manipulated variables	
	Liquid flow rate	Gas flow rate
Max width	Independent	Proportional
Length	Proportional	Proportional
Average speed	Inverse	Proportional
Arm diameter	Proportional	Inverse
Max width *y* position	Inverse	Proportional
Sheet thickness	Proportional	Inverse
Thickness scaling exponent	Inverse	Proportional
