# Supplementary material for: Simple and robust aqueous sheet jets in vacuum
Source: IUCrJ. 2026 Sep 1;13(Pt 5):659–67. doi: 10.1107/S2052252526007013 (PMC13548828; doi:10.1107/S2052252526007013)
Supplement: Supplementary file 1 [file m-13-00659-sup1.pdf]

IUCrJ

Volume 13 (2026)

Supporting information for article:

Simple and robust aqueous sheet jets in vacuum

Patrick E. Konold, Adil Ansari, Daniel Westphal, Patrick Adams, Romain Letrun, Joana Valerio, Johan Bielecki, Roberto C. Alvarez, Szabolcs Bódizs, Katerina Dörner, Gabriel Ducrocq, Lukas Grunewald, Konstantin Kharitonov, Chan Kim, Marco Kloos, Anna Králová, Taru Larkiala, Michael Maihöfer, Dimitra Manatou, Robert Mehlig, Diogo V. M. Melo, Petra Meszaros, Leonardo Monrroy, Tokushi Sato, Robin Schubert, Peter Smyth, Egor Sobolev, Fabian Trost, Raphael de Wijn, Lena Worbs, Tej Varma Yenupuri, Tong You, Joachim Schulz, Richard Bean, Kartik Ayyer, María Agustina Domínguez Martin, Henning Kirst, Helmut Grubmüller, Sebastian Westenhoff, Richard A. Kirian and Filipe R. N. C. Maia

Supporting Information for Simple and Robust Aqueous Sheet Jets in Vacuum

Nozzle fabrication by 3D-Printing

ASU protocol

The Nanoscribe Professional GT instrument was used to fabricate liquid sheet nozzles. The IP-S photoresist was cured via two-photon polymerization using a laser power of 80% and a scan speed of 100 mm/s. Printing was performed in blocks measuring 285 µm × 285 µm × 200 µm with a stitching overlap of 2 µm. The effective voxel size was $(1\ \mu m)^2 \times (3\ \mu m)$. The structures were printed on a glass substrate coated with an indium tin oxide (ITO) conductive layer. After printing, the samples were immersed in SU-8 developer for 15 minutes, detached from the substrate using tweezers, and then further developed overnight on an orbital shaker rotating at 120 RPM. The SU-8 developer was subsequently replaced with isopropyl alcohol (IPA), and the nozzles were stored in 1.5 mL Eppendorf tubes containing IPA until assembly. For assembly, the nozzles were placed under a microscope on the edge of a polydimethylsiloxane (PDMS) slide, dried, and secured with Kapton tape to enable insertion and gluing of capillary connections using fast curing epoxy.

Uppsala protocol

Nozzles were produced using the NanoOne 3D-printing system (UpNano). Nozzle design was performed with the Solidworks computer-aided design (CAD) program (Dassault Systèmes). The resulting STL files were loaded in the instrument control software (Think3D) for configuration of printer parameters. The model was divided into 700 x 700 x 60 µm blocks and the printer was operated in coarse mode. TPP was initiated with a 800 nm laser (50 mW) focused with a 20x objective. This yielded a voxel size of 0.5 x 0.5 x 4.5 µm. The printing was carried out on a 10 x 10 x 5.5 mm glass substrate (silanized) in *vat* mode, where the glass surface was submerged in liquid resin (UpPhoto). Following printing, the completed nozzles were placed in a beaker and soaked in dibasic ester (Carl Roth Solvagreen) for ~24 hours while gently stirring to remove residual unpolymerized resin. Upon sufficient development, the

nozzles were washed with isopropanol and dried for future use. The nozzles were assembled as previously described.

Thin film interference equations

For water with 450 nm light, the refractive index n is around 1.337. For an incidence angle of $\pi/4$, the angle of refraction α is about 0.56 radians. According to the Fresnel equations, in these conditions for unpolarized light only about 2.75% of it gets reflected at the air/water interface. Given that the vast majority of the light is transmitted, we make the simplifying assumption that the amplitude of the wave reflected at the top interface is equivalent to the one reflected at the bottom interface. Furthermore, we disregard higher order reflection as their power is negligible.

The optical path length difference (OPD) of a ray reflecting from the bottom of the liquid sheet jet, versus the one reflecting from the top of the liquid sheet jet is given by:

$$OPD = 2\,nt\cos(\alpha) \qquad \text{(S.1)}$$

where n is the refractive index of the sheet, t its thickness and α the angle of refraction that the ray makes with the normal to the bottom of the liquid sheet jet (after refraction). Given the extra π phase shift that occurs at the top or bottom interface (depending on the polarization), the phase shift between the two rays, δ, is given by:

$$\delta = \frac{4\,\pi\,nt\cos(\alpha)}{\lambda} + \pi \qquad \text{(S.2)}$$

The electric field resulting from the interference of the two rays, assuming they have equal amplitude, is then:

$$E \propto \frac{1+\exp(i\delta)}{2} \qquad \text{(S.3)}$$

The resulting intensity is then:

$$R \propto \left|\frac{1+\exp(i\delta)}{2}\right|^2 = \sin^2(\delta/2) \qquad \text{(S.4)}$$

The local maxima of R, corresponding to the bright and dark fringes are then given by the root of the derivative of S.4:

$$\frac{\partial R}{\partial \delta} = 0 \Leftrightarrow \frac{\partial R}{\partial \delta} = \sin(\delta)/2 = 0 \Rightarrow \delta = \pi\,m \Leftrightarrow t = \frac{m\,\lambda}{4\,n\cos(\alpha)} \qquad \text{(S.5)}$$

where m is the fringe order and m=1 is the first (thinnest) bright fringe.

In a real experiment, we measure not only the reflected light but also some background, I_b, which was estimated by measuring reflected light outside the sheet jet.

$$p = R + I_b \qquad \text{(S.6)}$$

where p is the measured intensity. Between any two adjacent fringes the thickness can be derived from the measured intensity p, the thickness (given by S.5) and measured intensities at the fringes, which we'll denote t_m and p_m respectively for the mth fringe. Noting that the p depends on the t in a sinusoidal way, and that $\sin^2$ and sin both corresponds to sinusoids, just of different frequency, one gets that:

$$t(p) = \frac{(t_{m+1} - t_m)}{\pi} \sin^{-1}\left(2\left(\frac{p - p_m}{p_{m+1} - p_m}\right) - 1\right) + \frac{(t_{m+1} + t_m)}{2} \qquad \text{(S.7)}$$

The standard deviation of background noise σ_p, a combination poisson and electronic noise, can be can be propagated to estimate the error bounds on the thickness:

$$\sigma_{t(p)} = \left| \frac{2\sigma_p (t_{m+1} - t_m)}{\pi (p_{m+1} - p_m) \sqrt{1 - \left(\frac{2(p - p_m)}{p_{m+1} - p_m} - 1\right)^2}} \right| \qquad \text{(S.8)}$$

At $\pi/4$ incidence angle, ignoring the higher order reflection yields a less than 5% error in thickness measurement between t_0 and t_1 and furthermore the error is inversely proportional to m between t_m and t_{m+1}.

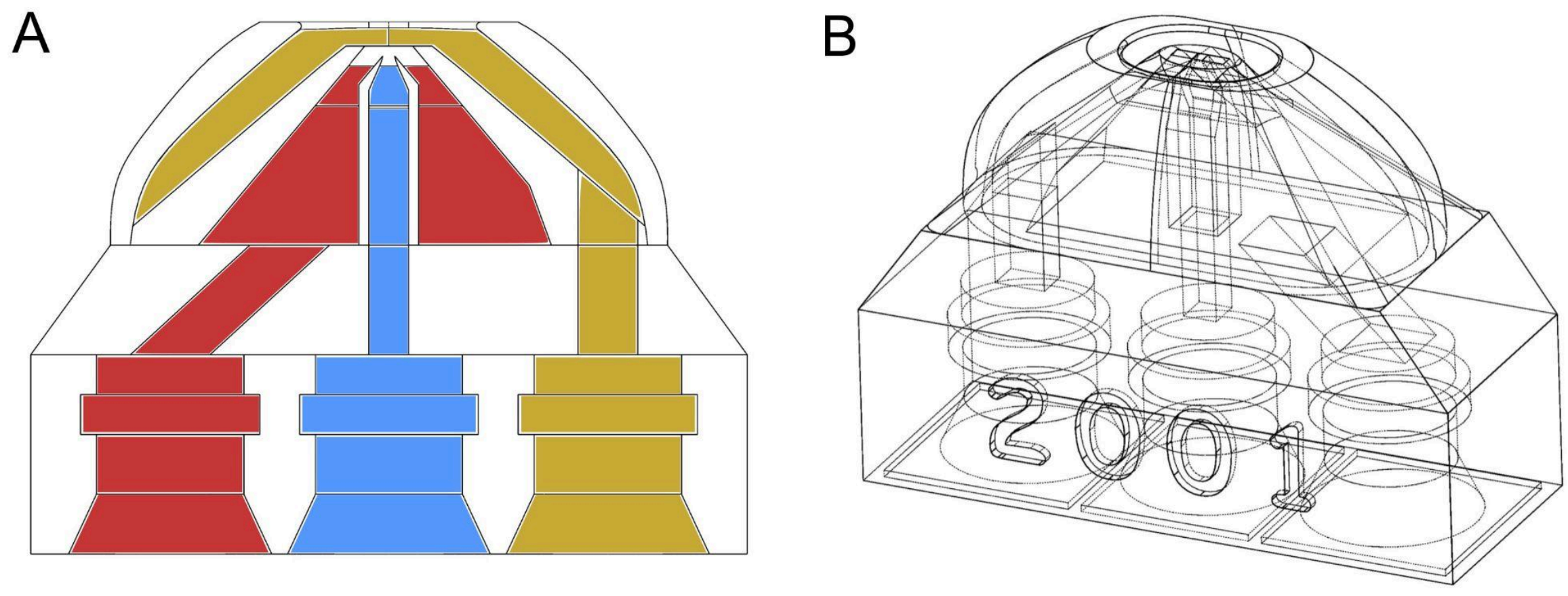

Supporting Figure 1. CAD drawings of the Uppsala GDSJ nozzle design. Internal channel regions for liquid (blue), impinging gas (red) and sheath gas (yellow) are noted.

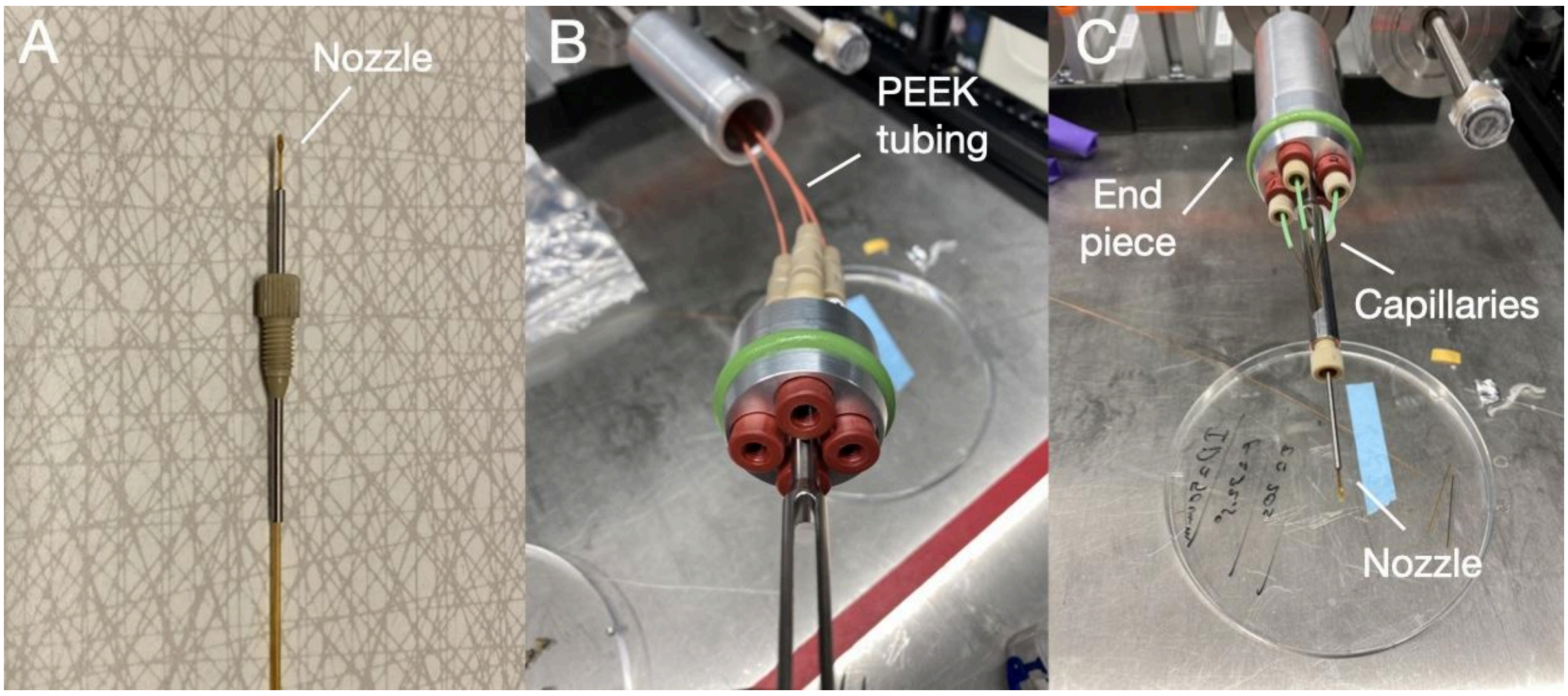

Supporting Figure 2. Modified liquid sheet jet nozzle mounting scheme at EuXFEL SPB/SFX. (A) 3D-printed nozzle with capillaries is first secured to a 1/16 inch stainless steel tubing with epoxy glue and a 10-32 conical PEEK fitting is positioned roughly 30 mm from the nozzle tip. (B) PEEK tubing run through the injector rod is connected to a custom built end piece. (C) Gas and liquid capillary connections are made to the end piece using 0.360 mm sleeve fittings. Finally, the nozzle is fixed to the end piece using the conical fitting in panel A.

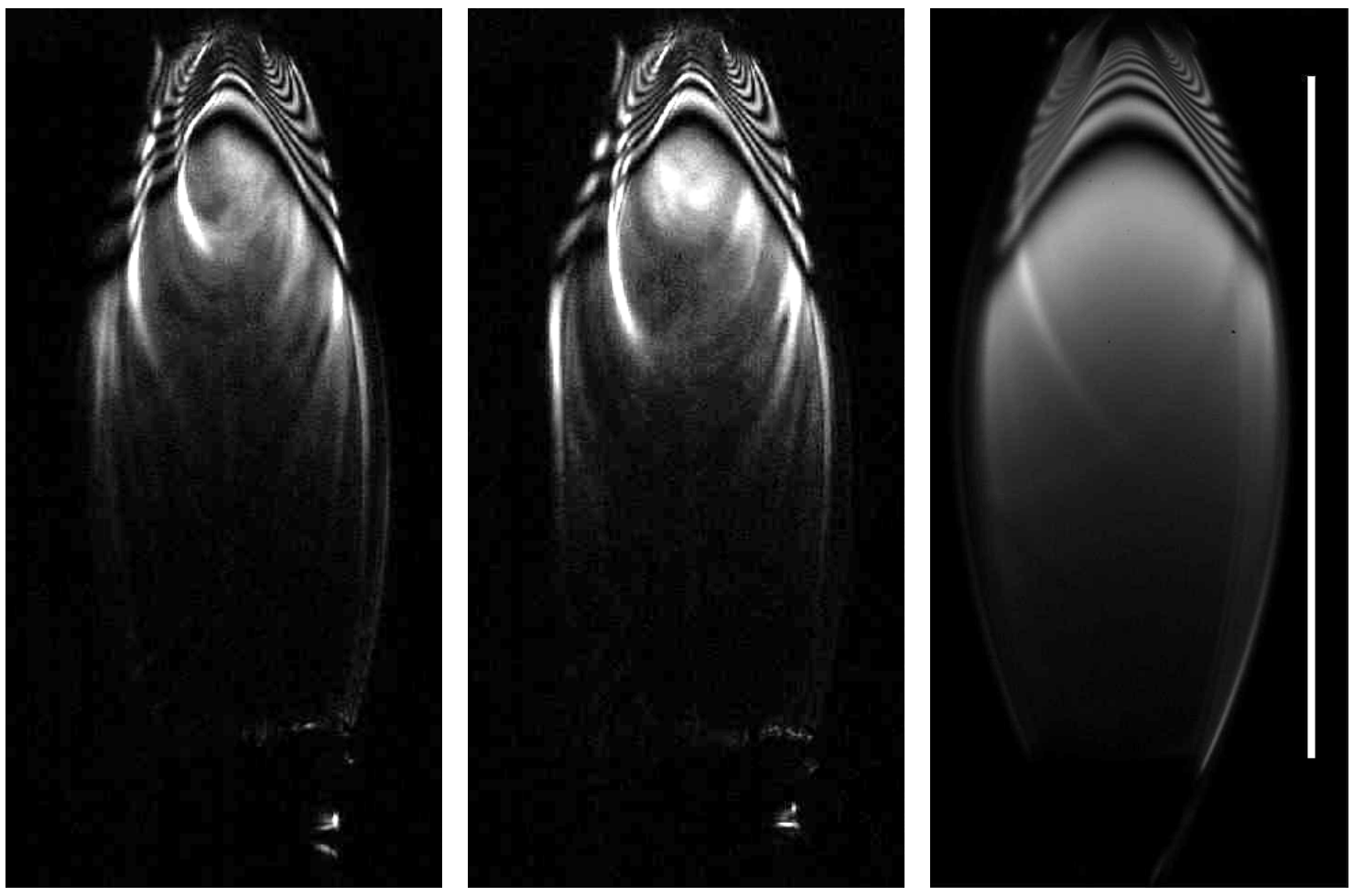

Supporting Figure 3. (left, center) Sheet reflection of two frames separated by 550 ns side illuminated by 650 nm 100 ns laser pulses. (right) Sheet reflection under continuous 450 nm blue diffused side illumination. The scale bar corresponds to 500 µm.

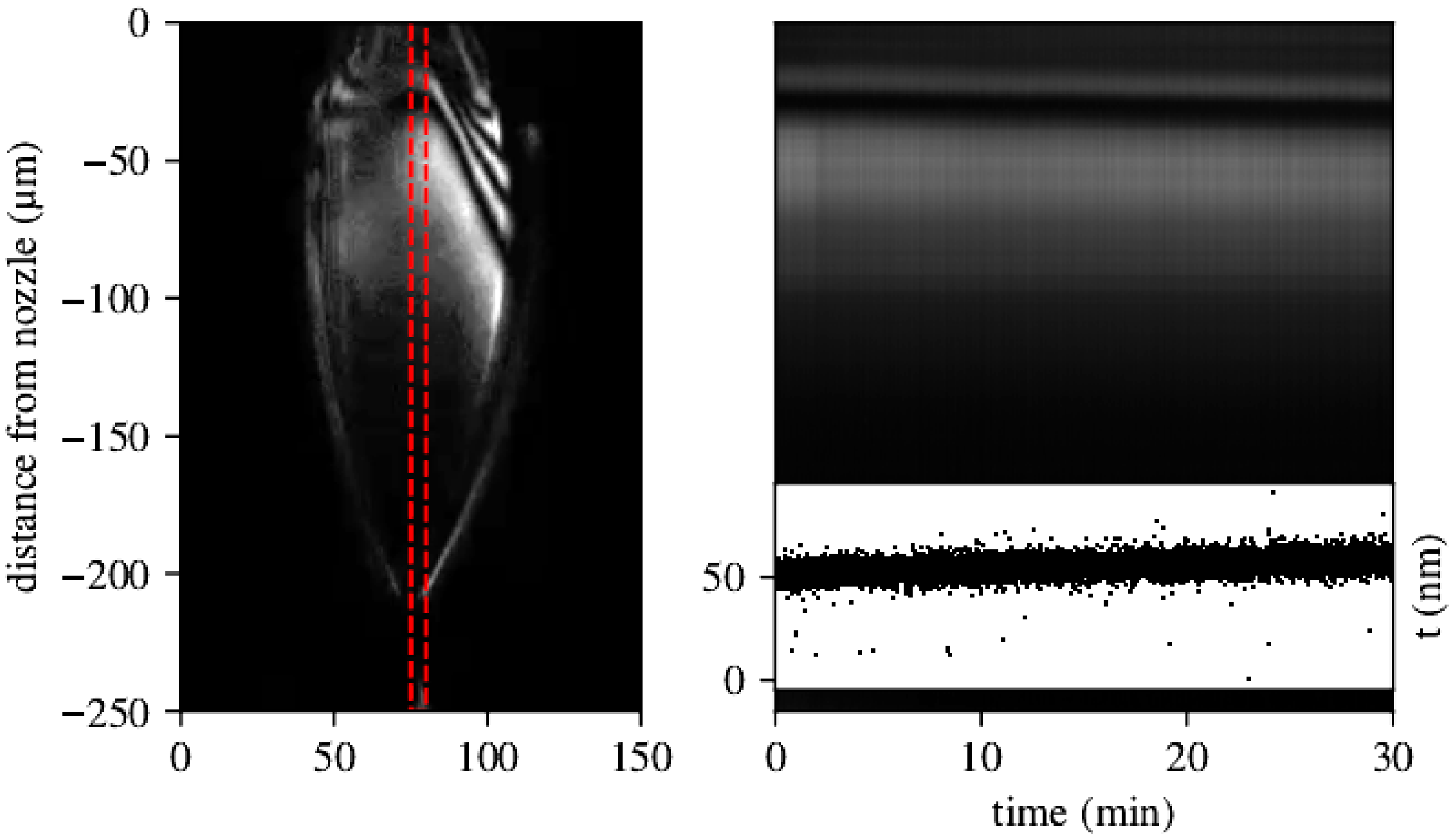

Supporting Figure 4. (Left) An aqueous sheet jet in vacuum with liquid flow rate of 150 μL/min and gas flow rate of 8 mg/min. (Right) Center line reflectance profile plotted over a period of half an hour together with the thickness at center of the sheet.

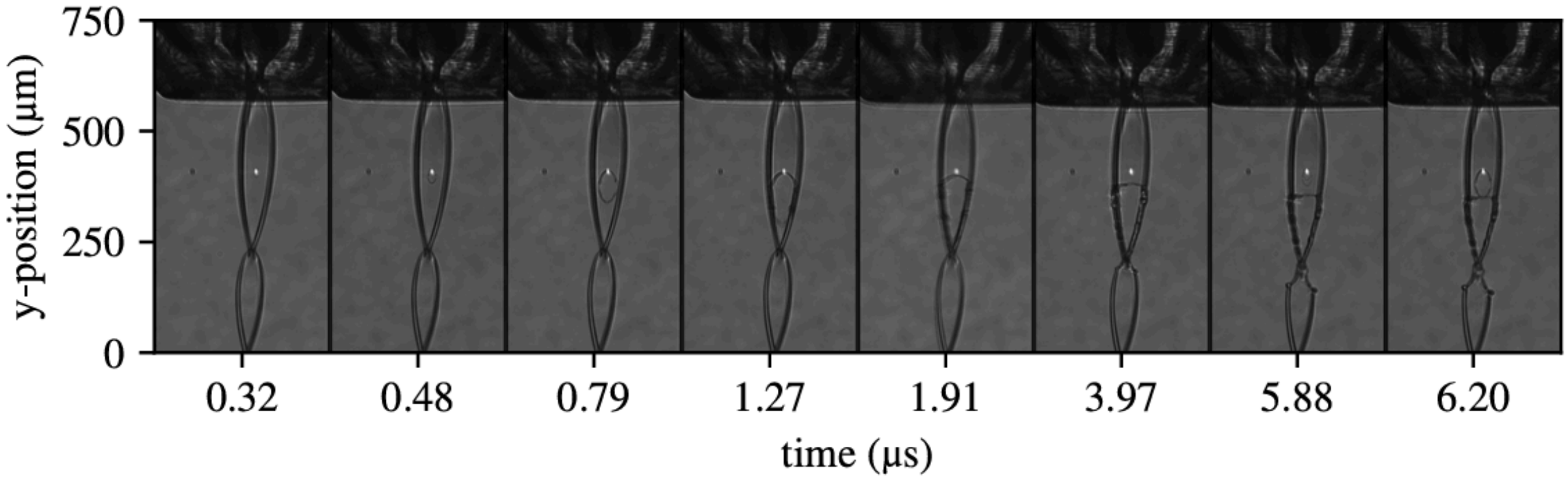

Supporting Figure 5. Jet explosion at EuXFEL SPB/SFX. Disruption of the sheet under a 256 kHz XFEL with a pulse energy of 2.5 mJ and photon energy of 6 keV with pulse duration of 15 fs. Each frame is a series of averaged images backlit with strobed femtosecond-pulsed 800 nm illumination laser at a fixed delay.

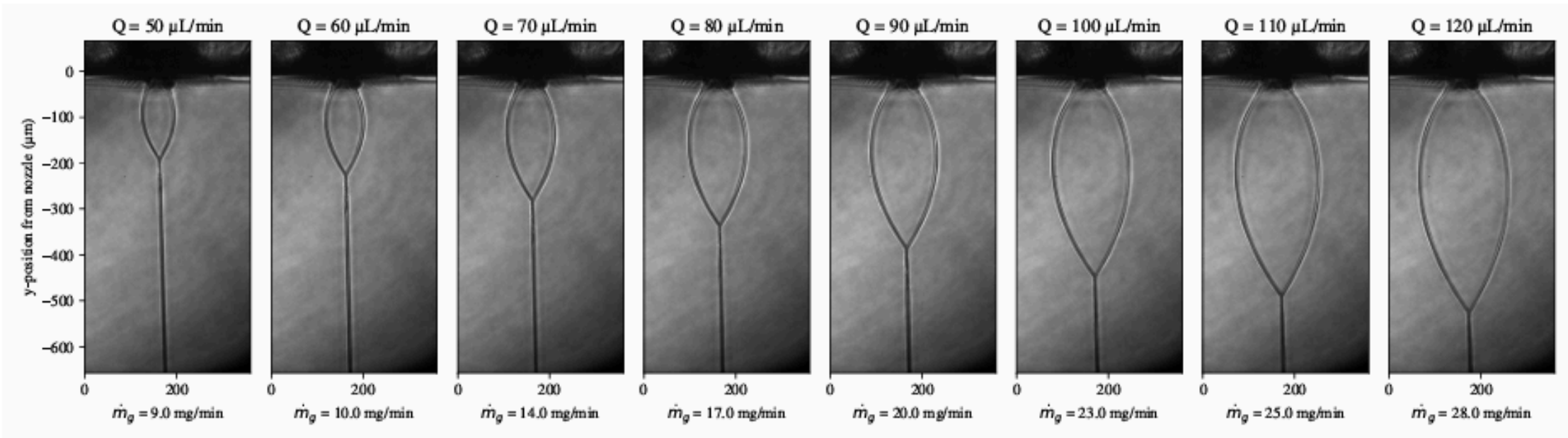

Supporting Figure 6. Effect of increased viscosity (40% v/v glycerol in water) and modified nozzle orifice area of (20 µm)^2 on sheet jet size. The resulting sheet length is nearly double that of an aqueous sheet jet produced with a (30 µm)^2 nozzle orifice at the same flow rate.

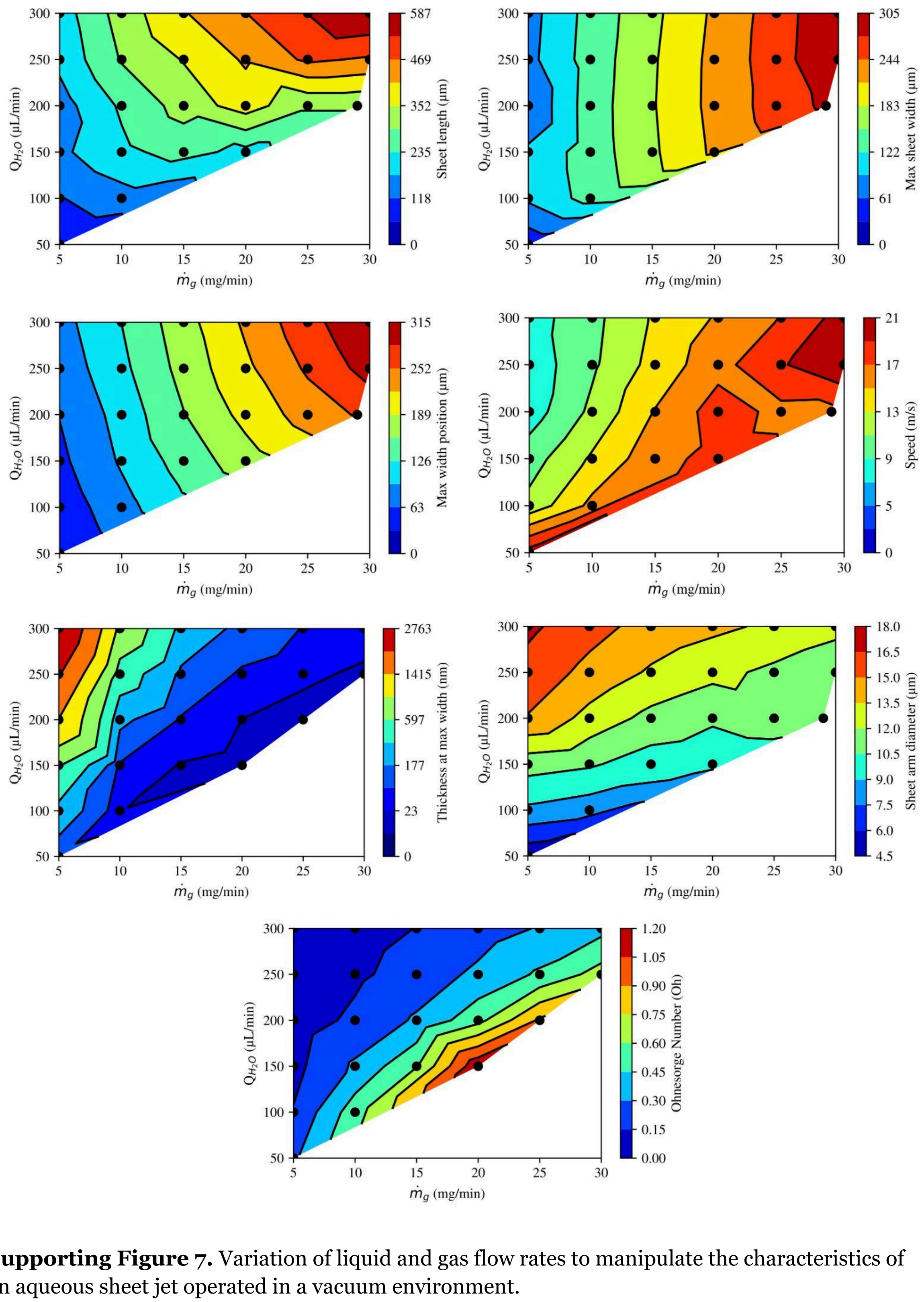

Supporting Figure 7. Variation of liquid and gas flow rates to manipulate the characteristics of an aqueous sheet jet operated in a vacuum environment.

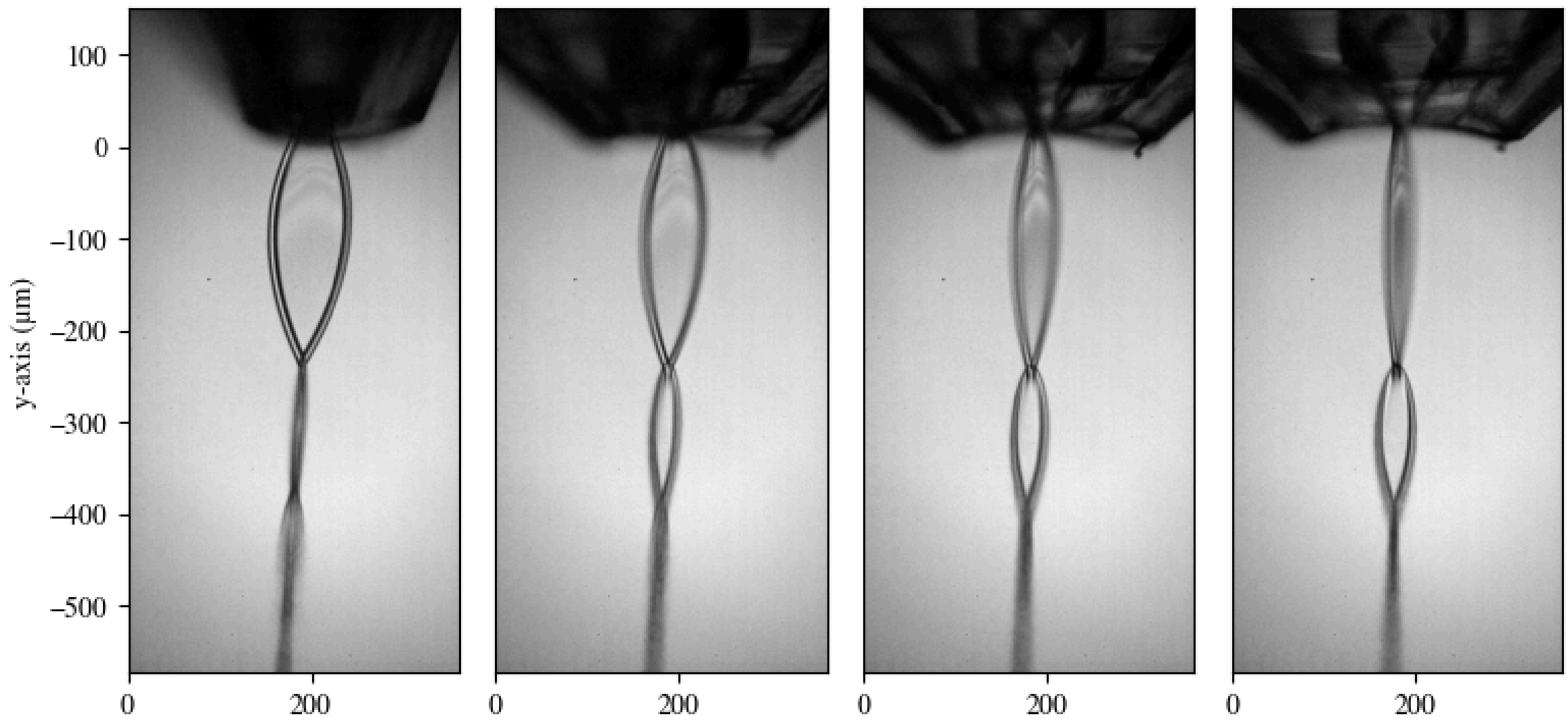

Supporting Figure 8. Angling the sheet jet perpendicular to the backlight direction improves fringe contrast according to Fresnel relationships.

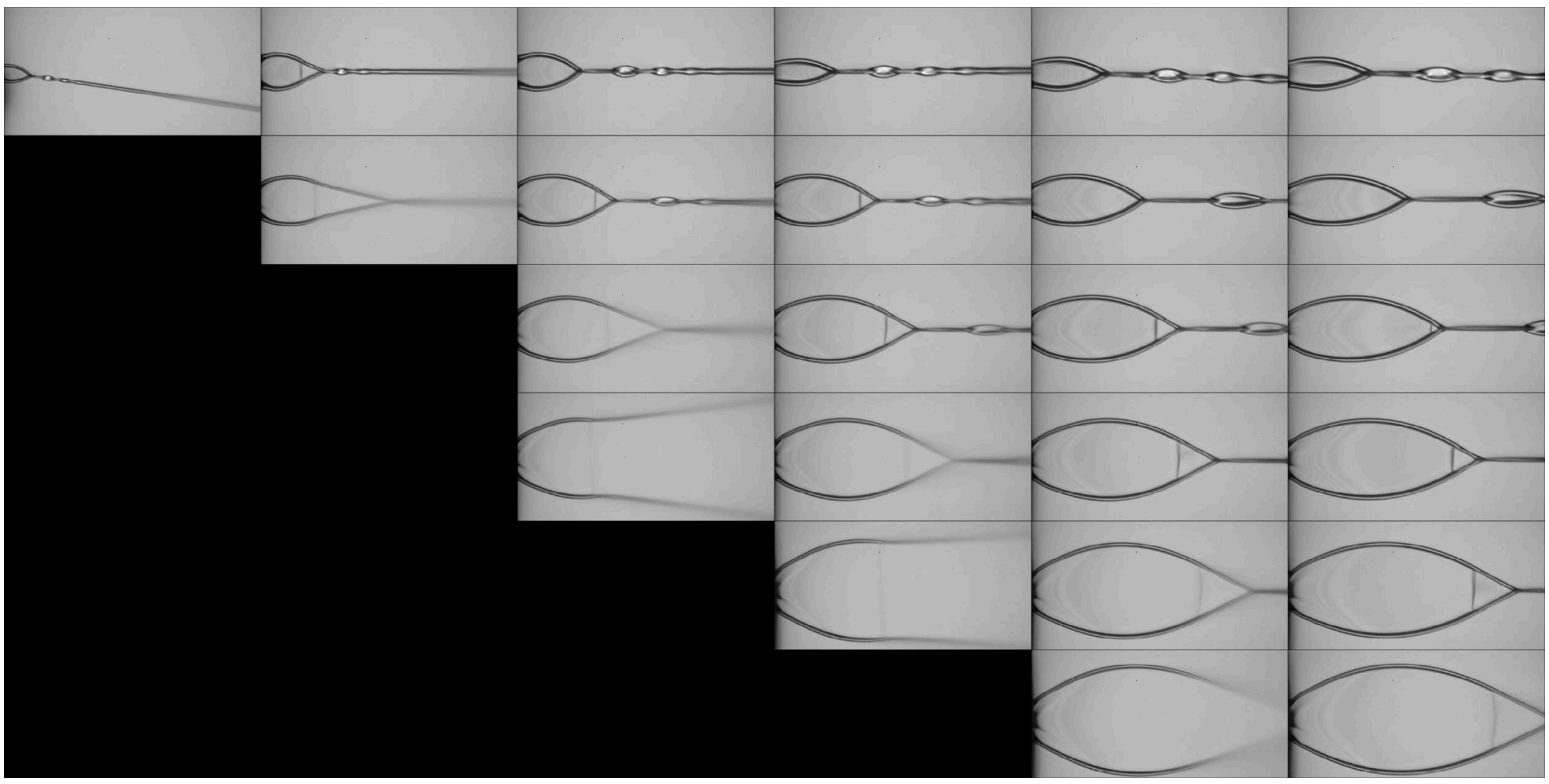

Supporting Figure 9. Images showing a 720 x 360 μm view of a sheet jet back-illuminated with 450 nm light. The horizontal sequence shows the liquid flow rate increasing from 50 to 300 μL/min in 50 μL/min increments from left to right. The gas flow rate increases from 5 to 30 mg/min in 5 mg/min increments from top to bottom.

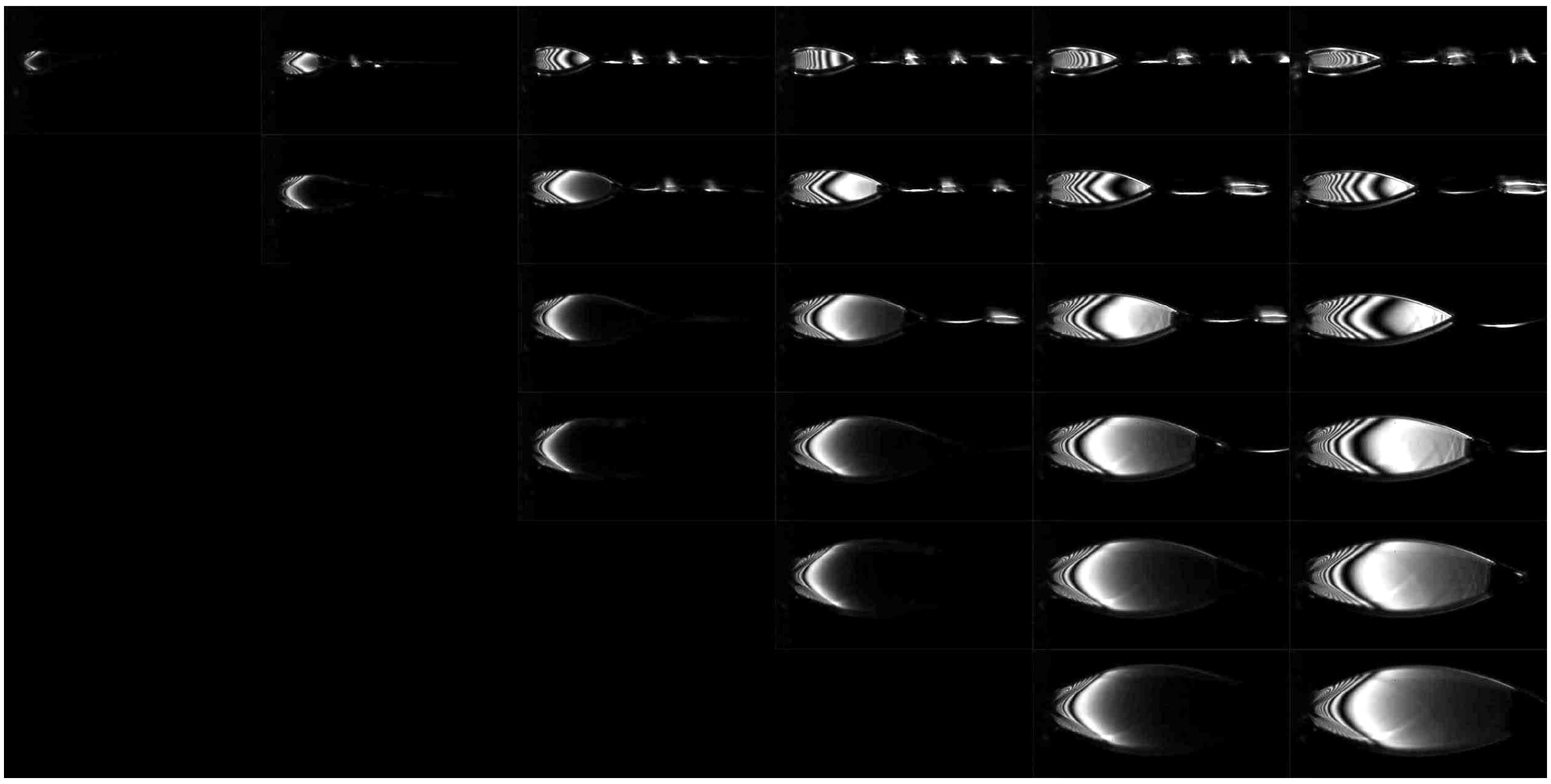

Supporting Figure 10. Same flow rate configurations as in Supporting Figure 9, but the sheet jet orientation is approximately 45° towards the camera.

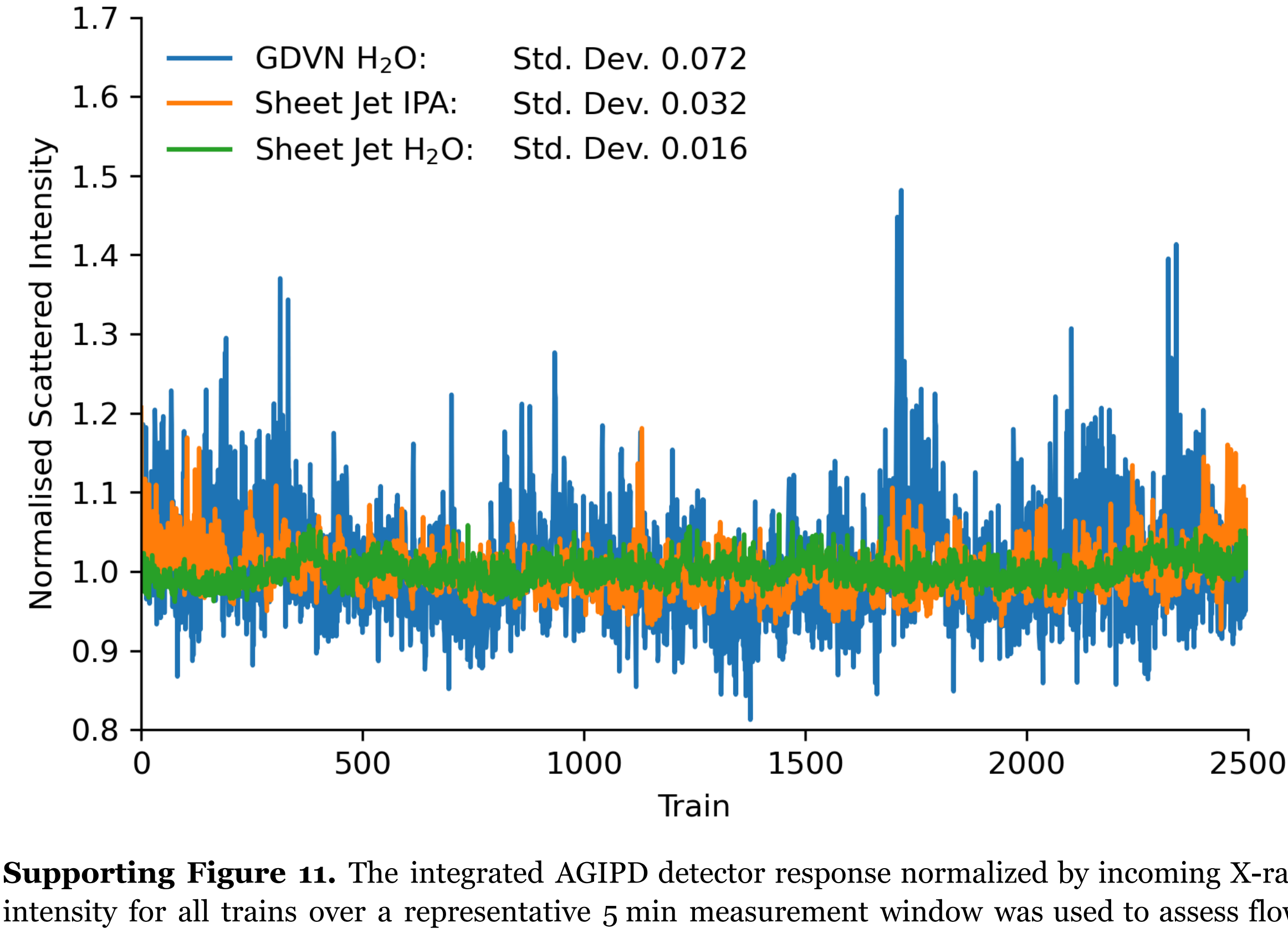

Supporting Figure 11. The integrated AGIPD detector response normalized by incoming X-ray intensity for all trains over a representative 5 min measurement window was used to assess flow stability among jets arising from different nozzle variants. The current (H2O) and our earlier (IPA) liquid sheet jet nozzles and a type B GDVN from the EuXFEL were compared. While both sheet jet nozzles outperformed the GDVN, our results indicate that the vacuum compatible version presented in this study yielded a 2-fold reduction in background fluctuations compared to our earlier design under similar experimental conditions.

Sheet explosion
Rim explosion

Supporting Figure 12. Stroboscopic movies of the intense XFEL pulse interaction with the liquid sheet jet (a) rim and (b) sheet regions. A shockwave with velocity of 1620 m/s is visible in the rim section following the arrival of the XFEL pulse.

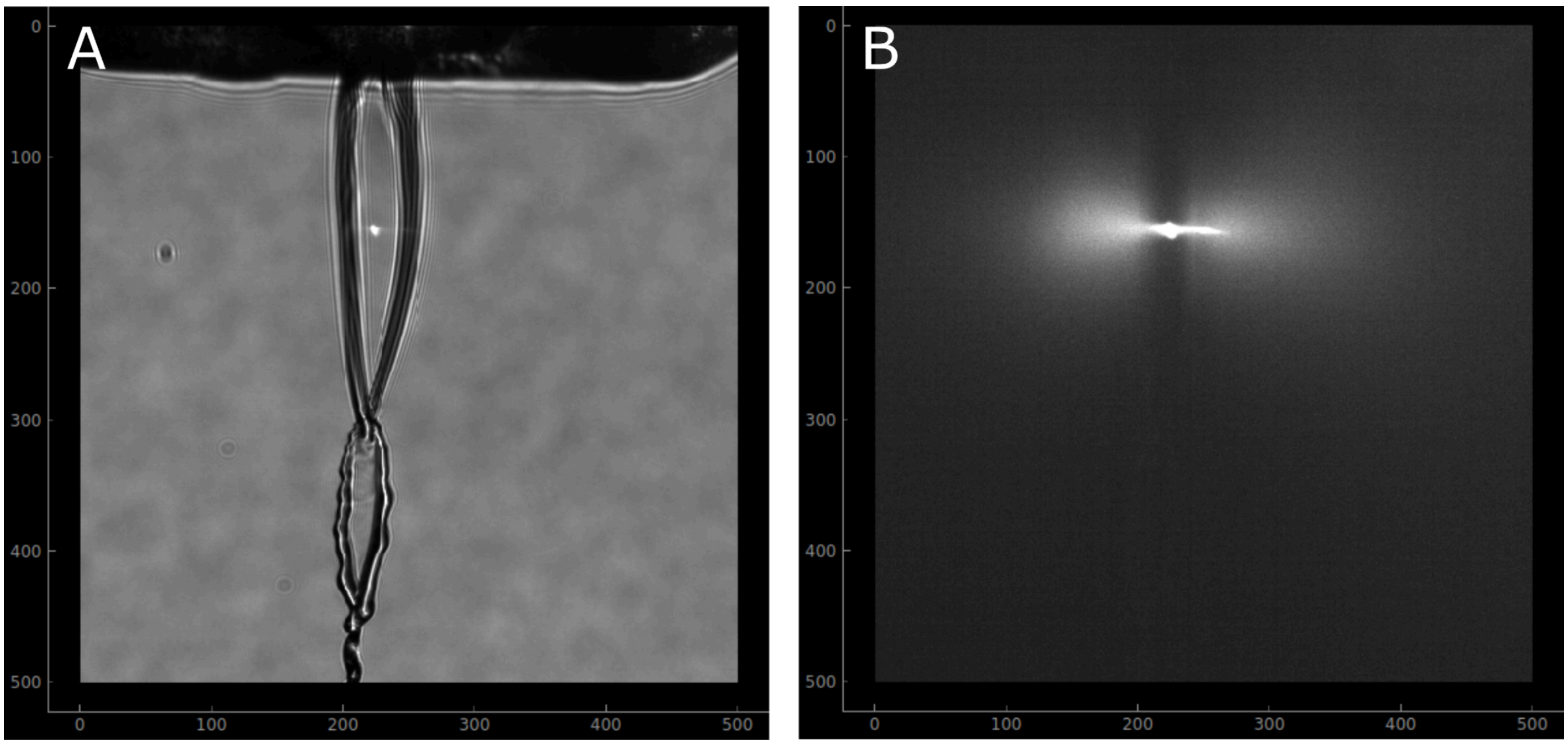

Supporting Figure 13. Snapshot of the liquid sheet jet (A) with and (B) without the femtosecond illumination laser. We attribute the glowing feature in panel B to the plasma generated upon exposure of the ultrathin liquid sheet with the XFEL pulse.
